# cGMP-Dependent Protein Kinase Type I Is Implicated in the Regulation of the Timing and Quality of Sleep and Wakefulness

**DOI:** 10.1371/journal.pone.0004238

**Published:** 2009-01-21

**Authors:** Sonja Langmesser, Paul Franken, Susanne Feil, Yann Emmenegger, Urs Albrecht, Robert Feil

**Affiliations:** 1 Division of Biochemistry, Department of Medicine, University of Fribourg, Fribourg, Switzerland; 2 Center for Integrative Genomics, University of Lausanne, Lausanne, Switzerland; 3 Interfakultäres Institut für Biochemie, Universität Tübingen, Tübingen, Germany; University of Maryland, United States of America

## Abstract

Many effects of nitric oxide (NO) are mediated by the activation of guanylyl cyclases and subsequent production of the second messenger cyclic guanosine-3′,5′-monophosphate (cGMP). cGMP activates cGMP-dependent protein kinases (PRKGs), which can therefore be considered downstream effectors of NO signaling. Since NO is thought to be involved in the regulation of both sleep and circadian rhythms, we analyzed these two processes in mice deficient for cGMP-dependent protein kinase type I (PRKG1) in the brain. *Prkg1* mutant mice showed a strikingly altered distribution of sleep and wakefulness over the 24 hours of a day as well as reductions in rapid-eye-movement sleep (REMS) duration and in non-REM sleep (NREMS) consolidation, and their ability to sustain waking episodes was compromised. Furthermore, they displayed a drastic decrease in electroencephalogram (EEG) power in the delta frequency range (1–4 Hz) under baseline conditions, which could be normalized after sleep deprivation. In line with the re-distribution of sleep and wakefulness, the analysis of wheel-running and drinking activity revealed more rest bouts during the activity phase and a higher percentage of daytime activity in mutant animals. No changes were observed in internal period length and phase-shifting properties of the circadian clock while chi-squared periodogram amplitude was significantly reduced, hinting at a less robust oscillator. These results indicate that PRKG1 might be involved in the stabilization and output strength of the circadian oscillator in mice. Moreover, PRKG1 deficiency results in an aberrant pattern, and consequently a reduced quality, of sleep and wakefulness, possibly due to a decreased wake-promoting output of the circadian system impinging upon sleep.

## Introduction

Life evolved in an environment of periodic recurrence of light and darkness. These steady changes have led to the incorporation of daily biological rhythms in order to schedule biochemical processes to their optimal phase during the 24 hours of a day. In mammals, the day can roughly be divided into an activity phase, during which physical activity is predominant, and a rest phase, during which repair mechanisms are activated and brain function alters into a state of sleep. Sleep is mainly controlled by two mechanisms: A homeostatic component regulates need and intensity of sleep according to the time spent awake or asleep, whereas a circadian component schedules sleep and wakefulness to the appropriate times within one day [1]. For the homeostatic process, a reliable index is provided by the amplitude and prevalence of delta (1 to 4 Hz) oscillations in the EEG of NREMS, also termed delta power. Delta power is high at the onset of sleep and consecutively decreases as animals rest. Sleep deprivation induces a predictable increase in delta power during subsequent sleep. For the circadian process in rodents, reliable information on the internal period length of the autonomous clock mechanism and the ability of the clock to adapt to changes in lighting schedules can be obtained by recording wheel-running activity. However, deciphering the molecular base of sleep is difficult because the contributions of the homeostatic and circadian processes are not easy to separate.

Increasing evidence hints at an involvement of NO signaling in the regulation of sleep, especially in that of NREMS [2]–[4] and sleep homeostasis [5], [6]. Variations of brain NO levels during the sleep-wake cycle were observed in rats [7], [8], and plasma levels of cGMP, a second messenger downstream of NO (reviewed in [9]), were found to be elevated at night in humans [10]. Moreover, NO and cGMP have been suggested to be involved in the modulation of circadian rhythmicity [11]. We therefore decided to investigate whether the NO-cGMP signaling pathway is involved in sleep regulation.

Many effects of NO in the nervous system are mediated via cGMP, which may act through various intracellular receptors, among them a family of serine/threonine kinases, the cGMP-dependent protein kinases (PRKG, also abbreviated cGK or PKG; reviewed in [12]). PRKGs in mammals are encoded by two genes, *Prkg1* and *Prkg2*. Whereas PRKG*2* has been reported to play a role in night-to-day progression and phase shifting of the circadian clock [13], [14], PRKG1 has been implicated in synaptic plasticity and learning (reviewed in [15]). Interestingly, PRKG1 is also expressed in brain regions that are involved in the regulation of sleep and circadian rhythms, such as the suprachiasmatic and other hypothalamic nuclei [16]–[18]. Furthermore, in *C. elegans*, overexpression of *egl-4*, a gene encoding for a cGMP-dependent protein kinase, caused adult animals to stop moving and feeding, whereas a lack of *egl-4* reduced behavioral quiescence in these animals [19]. These results suggest that cGMP-PRKG signaling promotes lethargus, a sleep-like state, in *C. elegans*.

To characterize the influence of PRKG1 on sleep and circadian rhythmicity in mammals, we analyzed two different mouse models lacking functional PRKG1 in the brain. We observed an altered pattern of sleep and reduced delta power under baseline conditions, indicating decreased sleep need in *Prkg1* mutants. After sleep deprivation, the difference in delta power disappeared, while the rebound in sleep time was increased in mutant mice. Furthermore, they displayed increased daytime activity and slower adaptation to alterations of the light-dark cycle pointing at a weak circadian oscillator. Taken together, our findings indicate that cGMP signaling via PRKG1 plays an important role in mammalian sleep regulation and timing of physical activity over the 24 hours of a day.

## Results

**Figure 1:**
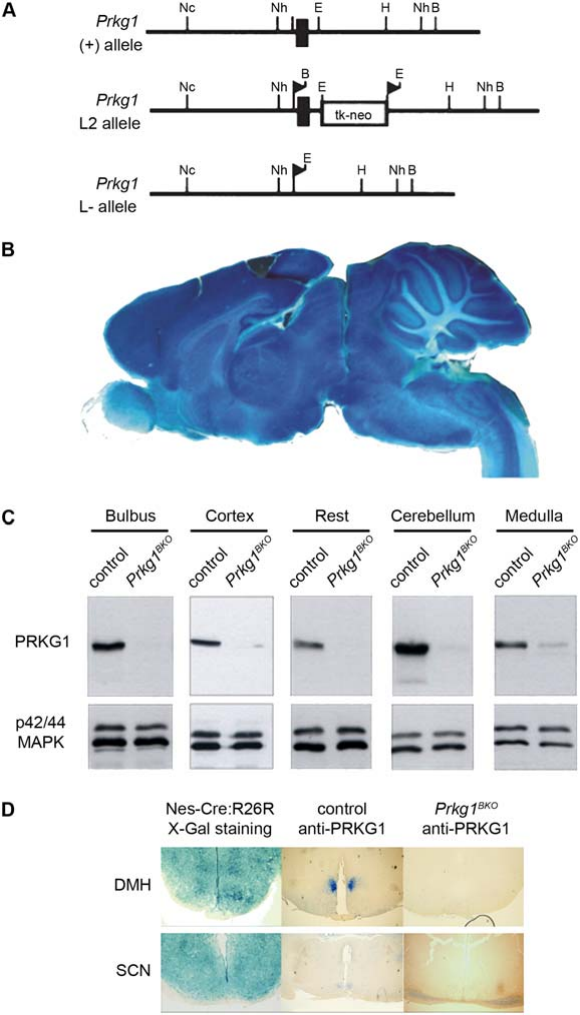
Generation of *Prkg1^BKO^* mice. (A) Strategy for conditional disruption of the murine *Prkg1* gene. Depicted are the *Prkg1* wild-type locus (+), the loxP-flanked conditional *Prkg1* allele (L2), and the excised *Prkg1* null allele (L−) obtained after Cre-mediated recombination of the L2 allele. The filled box denotes exon 10 of the murine *Prkg1* gene that encodes part of the ATP-binding site. Filled triangles indicate loxP sequences. The open box represents a thymidine kinase (tk)-neomycin resistance (neo) fusion gene. Restriction sites for *BamHI*, *EcoRI*, *HindIII*, *NcoI*, and *NheI* are indicated by B, E, H, Nc, and Nh, respectively. (B) X-Gal staining of a brain (sagittal view) of Nes-Cre;R26R reporter mice for Cre activity. (C) Western blot analysis of PRKG1 expression in various brain regions of control and *Prkg1^BKO^* mutant mice. The p42/44 mitogen-activated protein kinase (MAPK) was used as loading control. (D) Cre activity (left panels) and PRKG1 expression (middle and right panels) in the dorsomedial hypothalamus (DMH; upper panels) and suprachiasmatic nucleus (SCN, lower panels). Cre activity was monitored by X-Gal staining of cryosections of brains from Nes-Cre;R26R reporter mice. PRKG1 was detected by immunohistochemistry on paraffin sections of brains from control mice (middle panels) and *Prkg1^BKO^* mice (right panels).

A complete *Prkg1* knock-out leads to premature death at approximately 6 weeks of age, presumably due to smooth muscle dysfunction [20]. We therefore used two different conditional mouse models lacking *Prkg1* in the nervous system. In one model, *Prkg1* expression was rescued only in smooth muscle (SM) cells of *Prkg1* null mutants (*Prkg1^SMr^* mice) [21]. The other model was generated by Cre/lox-mediated neuron-specific inactivation of the *Prkg1* gene using the Nes-Cre line [22]; these mouse mutants were termed *Prkg1* brain knock-out mice (*Prkg1^BKO^* mice; see Fig. 1A and methods section). The recombination properties of Nes-Cre mice were confirmed by crossing them to R26R Cre reporter mice [23] and subsequent X-Gal staining of the brain for Cre activity (Fig. 1B). Western blot analysis (Fig. 1C) and immunohistochemistry (Fig. 1D) demonstrated an absence, or drastic reduction, of PRKG1 expression in various brain regions of *Prkg1^BKO^* mice. Since there are many brain regions involved in sleep regulation and the response to sleep deprivation (reviewed in [4]), *Prkg1^SMr^* mice, in which there is no risk of residual PRKG1 expression in any part of the nervous system, were chosen for sleep analysis. Circadian behavior, however, was studied in both lines. Circadian rhythms in overt behaviors are almost exclusively controlled by the suprachiasmatic nuclei (SCN). Therefore, we confirmed Cre activity in the SCN by X-Gal staining of R26R reporter mice and abolition of PRKG1 expression in the SCN of *Prkg1^BKO^* mice by immunohistochemistry (Fig. 1D) and consequently used also these mutants to assess the influence of PRKG1 on circadian rhythmicity.

### *Prkg1^SMr^* mice show a re-distribution of sleep and wakefulness

**Figure 2:**
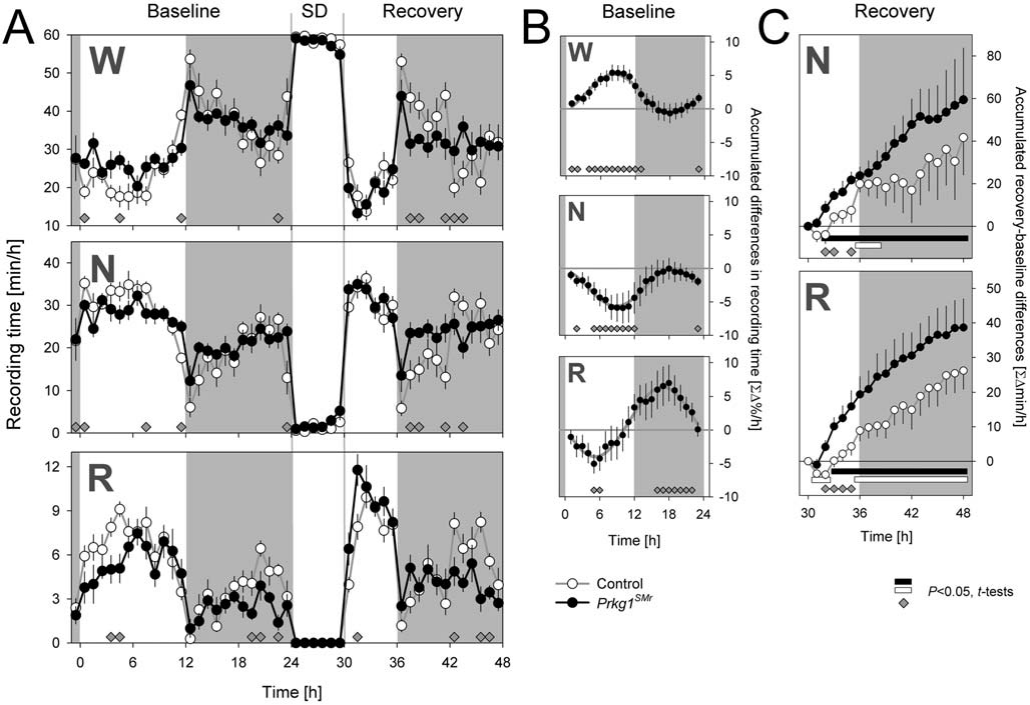
Amount and distribution of wakefulness, NREMS, and REMS during baseline and recovery from sleep deprivation. (A) Time course of mean (±1 SEM) number of minutes spent in each state for consecutive 1 h intervals (*Prkg1^SMr^*: filled symbols; control: open symbols) for the 48 h of the experiment. 0–24 h, baseline; 24–30 h, sleep deprivation; 30–48 h, recovery; W, wakefulness; N, NREMS; R, REMS; SD, sleep deprivation. (B) Summary of genotype differences in the relative distribution of the three behavioral states during baseline. Hourly, integrated values are first expressed as % of the total 24 h amount of each state within individuals (see Methods). Mean (±1 SE of the difference) *Prkg1^SMr^* – control differences in % accumulated are depicted. Gray lines represent fitted sine functions. Genotype affected the baseline time course of W and N but not of R [2-way ANOVA with factors ‘genotype’ (*P* = 0.41, 0.89, and 0.010) and ‘time’ (0–24; repeated measures: *P*<0.0001) and their interaction (*P* = 0.0012, 0.0007, and 0.064); *P*-values for W, N, and R, respectively]. (C) Recovery time course of time spent asleep lost during the 6 h SD. Hourly values were first expressed as differences (in min) between corresponding intervals during baseline (30–48 h vs. 6–24 h) within individuals, and then accumulated (see Methods). Values represent mean (±1SEM) accumulation curves for each genotype for N (top) and R (bottom panel). SD affected the amount and time course of time spent asleep [3-way ANOVA with factors ‘genotype’ (*P* = 0.24 and 0.25), ‘SD’ (recovery vs. baseline; repeated measures; *P* = 0.0054 and <0.0001) and ‘time’ (6–24 vs. 30–48; repeated measures: *P*<0.0001); interactions ‘genotype’×‘SD’ (*P* = 0.56 and 0.24), ‘genotype’×‘time’ (*P* = 0.0009 and 0.0001), ‘SD’×‘time’ (*P* = 0.042 and <0.0001); *P*-values for N and R, respectively]. Filled and open bars at the bottom of each panel connect intervals for which accumulated sleep time differed from baseline for *Prkg1^SMr^* and control mice, respectively (*P*<0.05; post-hoc 2-sided paired *t*-tests). In all panels, gray areas denote the dark periods and gray diamonds indicate 1 h intervals in which values significant differed between genotypes (*P*<0.05; post-hoc 2-sided *t*-tests).

Although *Prkg1^SMr^* mice did not differ in total time spent awake or asleep, the distribution of sleep and waking over the baseline day was markedly altered compared to control mice (Table 1, Figs. 2A, B). Mice are usually more active and awake during the dark as compared to the light period. The amplitude of this light-dark difference in sleep amount was significantly reduced in *Prkg1^SMr^* mice (Table 1). In comparison to control animals, *Prkg1^SMr^* mice were awake more in the first 7 h of the baseline light period and slept more during the hour preceding dark onset and the three subsequent hours (Figs. 2A, B). This pattern resulted in a sinusoidal time course of differences in accumulated time spent awake (Fig. 2B).

**Table 1:** Time-spent awake and asleep in baseline.

	Genotype	W	NREMS	REMS
24 h	control	12.17 (0.57)	9.78 (0.51)	2.05 (0.08)
	*Prkg1^SMr^*	12.72 (0.29)	9.70 (0.25)	1.58 (0.13)*
12 h light	control	4.60 (0.17)	6.05 (0.15)	1.35 (0.04)
	*Prkg1^SMr^*	5.26 (0.23)*	5.64 (0.20)	1.10 (0.09)*
12 h dark	control	7.57 (0.40)	3.73 (0.36)	0.69 (0.05)
	*Prkg1^SMr^*	7.46 (0.11)	4.06 (0.12)	0.48 (0.05)*
L-D difference	control	−2.98 (0.24)	2.31 (0.22)	0.66 (0.04)
	*Prkg1^SMr^*	−2.19 (0.22)*	1.57 (0.22)*	0.62 (0.06)

Mean (SEM; n = 7) time in hours spent awake (W), in NREMS, and REMS during baseline. Values were averaged over the entire day (24 h) and for the two lighting conditions separately (12 h light and 12 h dark). The light-dark (L-D) differences were calculated as well. Genotype affected the 24 h values of REMS and the LD distribution of W and NREMS [2-way ANOVA with factors ‘genotype’ (*P* = 0.41, 0.89, and 0.010) and ‘LD-condition’ (repeated measures: *P*<0.0001) and their interaction (*P* = 0.034, 0.033, and 0.58, for W, NREMS, and REMS, respectively)]. Asterisks indicate significant differences between genotypes (*P*<0.05; post-hoc 2-sided *t*-tests).

Lack of *Prkg1* did not equally affect NREMS and REMS. Although NREMS time did not differ between genotypes, *Prkg1^SMr^* mice spent less time in REMS than controls (Table 1, Fig. 2A). This reduction was present in both lighting conditions and the light-dark difference was not affected by genotype (Table 1). An analysis of the relative distribution of REMS over the 24 h baseline day (i.e., the percentage of each animal's total REMS during baseline) revealed, however, a redistribution also for this state (Fig. 2B). *Prkg1^SMr^* mice displayed less REMS in the first half of the light period, a deficit that was entirely compensated in the second half and, conversely, showed more REMS in the first half of the dark period, a surplus that was again lost in the second half. In contrast the effects on NREMS were the mirror image of those described above for wakefulness to a large extent (Table 1, Figs. 2A, B).

### Rebound sleep is increased in *Prkg1^SMr^* mutants

We next wanted to determine whether homeostatic regulation of sleep was altered in *Prkg1^SMr^* mice. Lack of *Prkg1* was found to affect the sleep recovery pattern after sleep deprivation, especially within the first 5 h (Fig. 2C; compare 30–35 h of recovery to 6–11 h of baseline). Over this period, *Prkg1^SMr^* mice accrued a 2.9-fold increase in extra NREMS time and a 3.7-fold increase in extra REMS time compared to control mice. Moreover, in *Prkg1^SMr^* mice rebound sleep was apparent already after 2 h whereas in control mice, sleep time was initially reduced and only after 6 h did accumulated values exceed baseline (Fig. 2C). In the dark period, these differences between genotypes persisted although significance levels were no longer reached.

### *Prkg1^SMr^* mice display a marked decrease in EEG delta power

**Figure 3:**
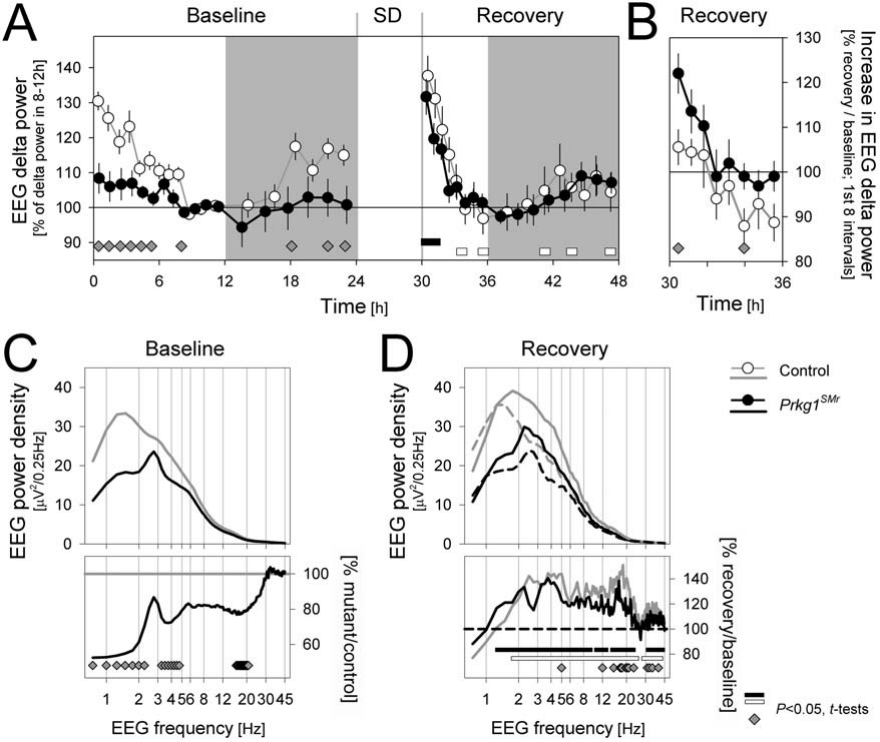
Time course of EEG delta power and EEG spectra during NREMS. (A) Time course of mean (±1SEM) relative levels of EEG delta (1.0–4.0 Hz) power for the 48 h of the experiment (*Prkg1^SMr^*: filled symbols; control: open symbols). Values were expressed as % of the lowest levels reached in baseline (8–12 h; see Methods for details). The dynamic range of baseline changes in delta power was greatly reduced in *Prkg1^SMr^* mice compared to controls while during recovery from sleep deprivation (SD) this genotype difference disappeared [2-way ANOVA with factors ‘genotype’ (*P* = 0.0063 and 0.53) and ‘time’ (repeated measures: *P*<0.0001) and their interaction (*P* = 0.017 and 0.15); *P*-values for baseline and recovery, respectively]. Gray areas denote the dark periods. (B) Effect of SD on EEG delta power during the light period. The first 8 values after recovery sleep onset were expressed as % of the first 8 values after sleep (or light) onset in baseline within individual mice. Values represent means±1SEM. (C) Spectral composition of the NREMS EEG during baseline (upper panel; absolute values 0.75–45 Hz at 0.25 Hz resolution, lower panel: % difference between genotypes). EEG power density was markedly lower in *Prkg1^SMr^* than control mice in the low delta (0.75–2.25 Hz) frequency range and to smaller extend also in the high delta (3.25–4.75 Hz) and beta (16–21 Hz) ranges. [2-way ANOVA with factors ‘genotype’ (*P* = 0.0072) and ‘EEG frequency’ (repeated measures, 0.75–45 Hz: *P*<0.0001) and their interaction (*P* = 0.0009)]. EEG frequency was plotted on a logarithmic scale to better illustrate differences in the lower frequencies. (D) Upper panel: NREMS EEG spectra during the first interval after sleep onset after SD (solid lines; see A) and the corresponding time-of-day during baseline (dashed lines; see A interval 7). Lower panel: % recovery/baseline change (Black lines: *Prkg1^SMr^*, gray lines: control). In all panels, gray diamonds at the bottom indicate times at or frequency bins in which values significant differed between genotypes (*P*<0.05; post-hoc 2-sided *t*-tests). In Panels A and D filled and open bars connect intervals for which values differed from baseline for *Prkg1^SMr^* and control mice, respectively (*P*<0.05; post-hoc 2-sided paired *t*-tests).

The EEG power in the delta frequency range (1–4 Hz; i.e., EEG delta power) during NREMS is widely used to index sleep need and we therefore followed the dynamics of this variable throughout the 2-day experiment. Striking differences between genotypes were observed during baseline (Fig. 3A). *Prkg1^SMr^* mice showed unusually low levels of EEG delta power at sleep onset (i.e., light onset) when sleep need and EEG delta power in mice tend to be highest (see control mice in Fig. 3A). Values remained lower for most of the initial 6 h of the light period. During the dark (active) period, EEG delta power increased in control mice, consistent with sleep need building up when waking prevails, and again was importantly reduced in *Prkg1^SMr^* mice. While no overall differences in time spent awake were found (Table 1), *Prkg1^SMr^* mice were awake more in the 6 h preceding light onset (*Prkg1^SMr^* vs. control mice: 3.3±0.2 h vs. 2.8±0.1 h; *P* = 0.028; 2-sided *t*-tests; data not included in Fig. 2 but consistent with observations in 18–24 h). Together, these observations strongly suggest that *Prkg1* deficiency alters the relationship between time spent awake and subsequent EEG delta power. However, under conditions of enforced wakefulness this relationship seemed normalized: delta power levels reached after 6 h of sleep deprivation and their subsequent time course during recovery did not differ between genotypes (Fig. 3A). Relative delta power after sleep deprivation, expressed as percent of baseline values, was elevated in *Prkg1^SMr^* during the first recovery interval only (Fig. 3B).

### The quality of sleep and wakefulness is reduced in *Prkg1^SMr^* mice

The pronounced baseline differences in EEG delta power might be due to differences in the quality of waking impeding a build-up of sleep need. The spectral composition of the waking EEG did not greatly differ between the two genotypes. This was true for the entire 24 h baseline recordings (Fig. S1) as well as during the last 6 h of the dark period; i.e., the period preceding the time when genotype differences in EEG delta power were maximal (data not shown). When rats spent more of their waking time exploring, EEG delta power in subsequent NREMS was reported to be higher [24]. We therefore analyzed theta activity (4–8 Hz) in the waking EEG, which is characteristic of exploratory behavior. No differences between genotypes were observed in the frequency or peak amplitude of EEG theta activity during the last 6 h of the baseline dark period or in the duration and distribution of wakefulness with distinct theta activity (i.e., theta-dominated wakefulness; data not shown). One parameter that greatly differed between the two genotypes was, however, waking episode duration (Fig. S2). *Prkg1^SMr^* mice spent more of their waking time in shorter waking episodes (with durations between 16 s and 2 min) and substantially less time in long waking episodes. Whereas in control mice, episodes longer than 17 min contributed to 35% of the total time spent awake, in *Prkg1^SMr^* mice only 11% was spent in these longer waking bouts. Thus, the inability to sustain longer periods of waking might have contributed to a reduced build-up of sleep need in *Prkg1^SMr^* mice.

It also appears that *Prkg1* affects sleep quality. Fragmentation of NREMS can be quantified by counting the number of short (<60 s) NREMS episodes [25]. NREMS was severely fragmented in *Prkg1^SMr^* mice since they showed more short and less long episodes (short: 68.6±8.4 vs. 43.3±3.6; *P* = 0.016; long NREMS episodes: 17.7±0.8 vs. 19.8±0.3 *P* = 0.030; post-hoc 2-sided *t*-tests; all values expressed per hour of NREMS). Accordingly, the frequency distribution of NREMS episode duration was shifted towards shorter episodes in *Prkg1^SMr^* mice (Fig. S2). NREMS quality was also assessed by inspection of the spectral composition of the EEG during this state. EEG power density was markedly lower in *Prkg1^SMr^* than in control mice in the low delta (0.75–2.25 Hz) frequency range and to a lesser extent also in the high delta (3.25–4.75 Hz) and beta (16–21 Hz) ranges (Fig. 3C). The reduction in EEG power in the low delta frequency band persisted even after taking genotype differences in the overall EEG amplitude into account. No significant changes between genotypes were observed in the EEG spectra in REMS and wakefulness (Fig. S1).

### Slow and fast delta frequencies are not equally affected

An additional factor that might play a role in the baseline differences in EEG delta power is the 2-fold decrease in the EEG activity in the low delta frequencies (1.0–2.25 Hz) during NREMS observed in *Prkg1^SMr^* mice (see above). As a consequence of this, EEG delta power, calculated over the entire delta frequency range (1–4 Hz), might reflect sleep need less accurately. We therefore analyzed the sleep-wake dependent dynamics of EEG delta power for slow and fast delta frequencies separately (Fig. S3). For both genotypes the changes in the faster delta frequencies clearly respond more precisely to the changes in sleep and waking. This is especially obvious in the dark periods, when relative power in the slow delta frequencies was importantly lower than in the faster delta frequencies. Also during recovery from sleep deprivation, both the positive and subsequent negative rebound in EEG activity in the fast delta frequencies were lacking in the slow frequencies in control mice. Despite these changes, sleep deprivation affected EEG activity in the two delta bands similarly in the two genotypes, and the altered NREMS EEG spectra observed for *Prkg1^SMr^* mice during baseline were present also after sleep deprivation (Fig. 3D, see also the fast-to-slow delta power ratio in Fig. S3B).

### The amplitude of circadian rhythmicity is decreased in *Prkg1* mutant mice

**Figure 4:**
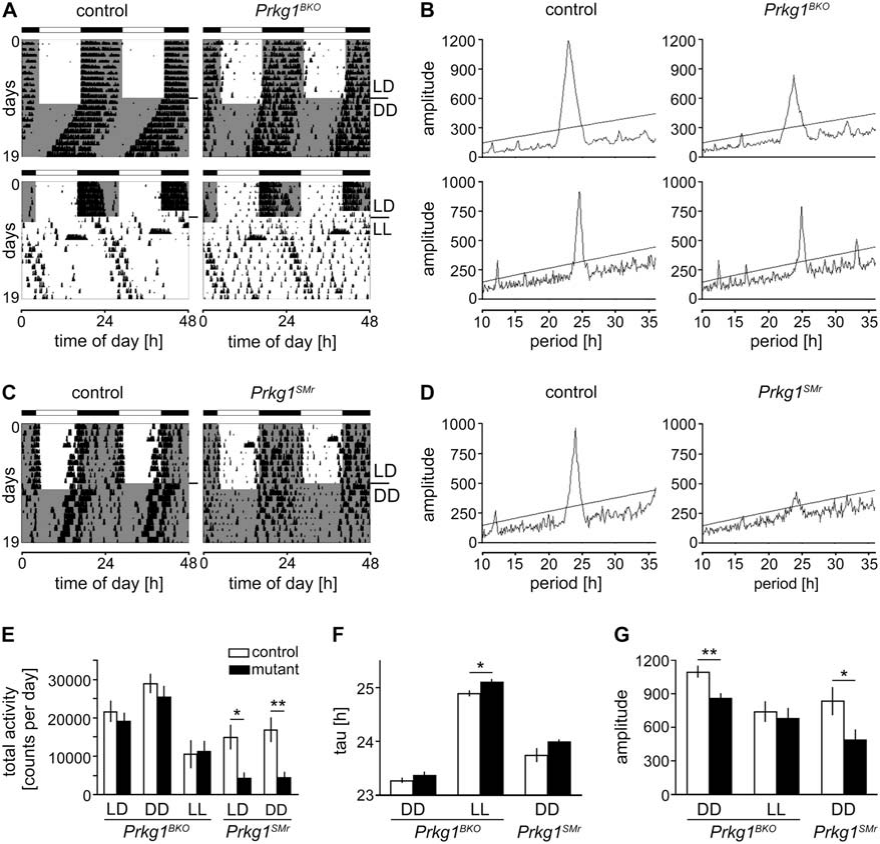
Circadian phenotype of *Prkg1^BKO^* and *Prkg1^SMr^* mutants. (A) and (C) Representative activity recordings (actograms) of *Prkg1^BKO^* (A) and *Prkg1^SMr^* (C) mutant mice and their respective controls whose wheel-running activity is plotted as vertical bars in a double-plot format. Each horizontal line represents two 24 h periods; the second (right) half of each line is repeated on the first (left) half of the following line. Mice were entrained to LD 12∶12 and subsequently released into DD (A, upper panels, and C) or LL (A, lower panels). Black and white bars on top depict the distribution of dark and light periods during initial LD, shaded areas in the actograms indicate actual dark periods during the experiment. LD, light-dark cycle; DD, constant darkness; LL, constant light. (B) and (D) Theoretical probability distributions for period lengths between 10 and 35 hours (chi square periodograms) of the activity data obtained under DD (B, upper panels, and D) or LL (B, lower panels) for the same animals whose actograms are shown in (A) and (C). The X coordinate of the peak indicates the internal period length of the free-running rhythm, the Y coordinate is a measure of the robustness of the rhythm. Note that Y axis scales are the same for adjoining periodograms to make differences more readily visible. (E)–(G) Total activity values (E), internal period length tau (F) and amplitude of chi square periodograms (G) for *Prkg1^BKO^* and *Prkg1^SMr^* mutants and their respective controls in LD (only for activity), DD and LL (only for *Prkg1^BKO^* mutants and controls). n = 18 for *Prkg1^BKO^* mutants, n = 20 for the respective controls for LD and DD; n = 9 for *Prkg1^BKO^* mutants, n = 11 for controls for LL; n = 6 for *Prkg1^SMr^* mutants and the respective controls. ** p<0.01, * p<0.05 as determined by unpaired t-test. LD, light-dark cycle; DD, constant darkness; LL, constant light.

Since re-distribution of sleep might be influenced by the circadian clock, we monitored wheel-running activity of *Prkg1^SMr^* and *Prkg1^BKO^* mutants as well as their respective controls under different lighting conditions. Animals were entrained to a 12 hours light/12 hours dark cycle (LD 12∶12) and subsequently released into constant darkness (DD) or constant light (LL). We observed that animals, as expected, were mostly active during the dark period in LD and that all genotypes were able to maintain circadian, i.e., rhythmic, wheel running activity in DD (Fig. 4A–D). Total activity under all lighting conditions tested was not different for *Prkg1^BKO^* mutants as compared to control animals whereas *Prkg1^SMr^* mutant mice showed drastically reduced activity both in LD and DD (Fig. 4E). *Prkg1^SMr^* mutants were therefore not subjected to LL since in LL, activity can be expected to be even lower. The reduced wheel running activity of *Prkg1^SMr^* mice was not due to a decreased general locomotor activity, since an open field test in *Prkg1^SMr^* mice revealed no differences in total distance, mean speed and percentage of time spent active between *Prkg1^SMr^* mice and controls (Fig. S4). This is in line with the observation that there is no difference in theta power and theta-dominated wakefulness between these two genotypes (see above).

Internal period length; i.e., tau, in DD did not differ between either mutant and its respective control, whereas a slight difference in the period lengths determined under LL conditions was observed for *Prkg1^BKO^* mutant and control mice (Fig. 4F). Chi square periodogram amplitude, which has been used as a measure of the robustness of circadian rhythms [26], [27], was found to be significantly lower in both mutant strains in DD, but not in LL in *Prkg1^BKO^* mutant mice (Fig. 4G). This hints at a possible role of PRKG1 in the stabilization of the circadian oscillator. In order to investigate this phenomenon in more detail, additional wheel-running experiments were performed. This time, we used exclusively *Prkg1^BKO^* mutant and control animals since they displayed overall higher chi square periodogram amplitudes than *Prkg1^SMr^* mutants and controls, indicating a more stable oscillator.

### *Prkg1^BKO^* mice show higher onset errors and reduced activity after chronic jet-lag

Since PRKG2 has been implicated in clock resetting [13], we tested whether *Prkg1^BKO^* animals, as compared to control mice, reacted differently to a nocturnal light pulse (Fig. S5). We observed no differences for light pulses applied at ZT14 or ZT22 (beginning and end of the subjective night, respectively), however. From this we conclude that PRKG1 probably does not play an important role in the acute light-signaling pathway that influences phase shifts of the circadian clock. The results obtained in LL conditions (see above) indicate, though, that PRKG1 might be implied in the integration of external light cues, and possibly in the determination of daylength since *Prkg1^BKO^* mice appear to have a longer circadian day under LL conditions (Fig. 4F).

**Figure 5:**
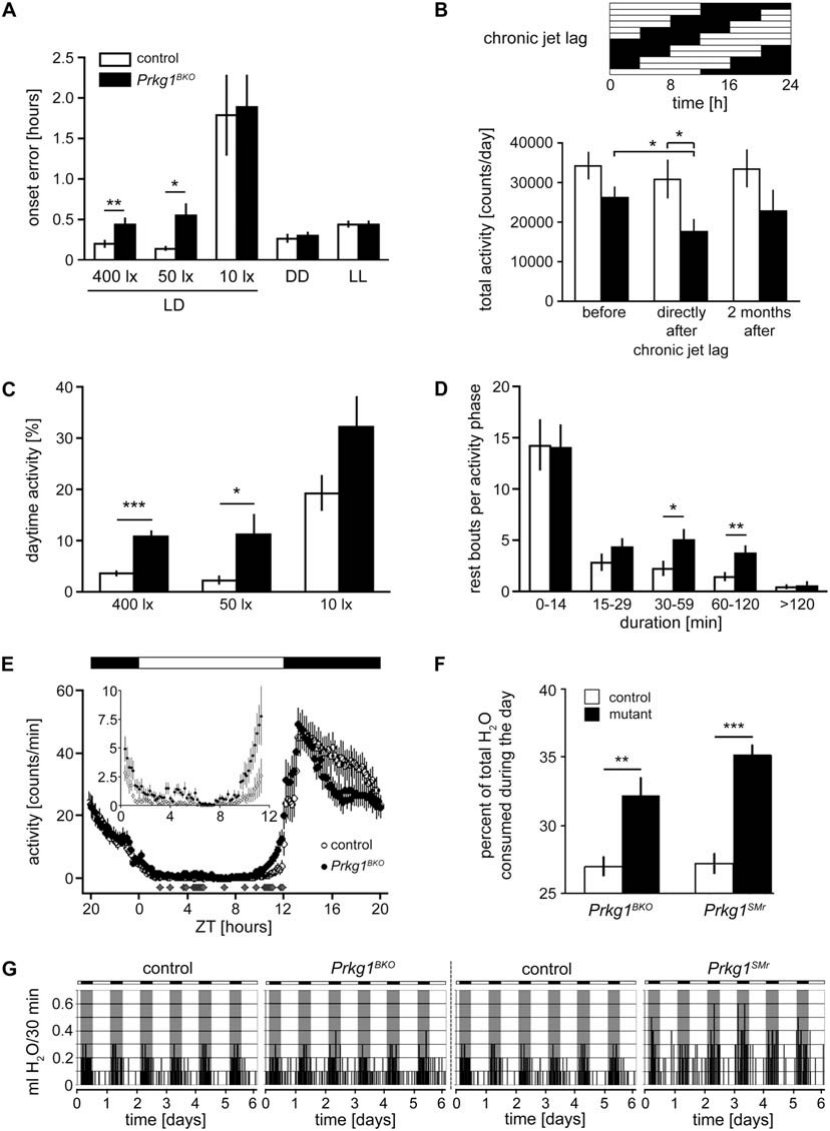
*Prkg1^BKO^* have a weakened circadian oscillator. (A) Onset errors of control and *Prkg1^BKO^* mutant mice kept in LD 12∶12 of the indicated intensities, DD and LL were determined from the actograms. They are expressed as the average deviation of the actual onset from lights off for LD and from a regression line drawn through all onsets for DD and LL. n = 5 for both genotypes for LD 50 and 10 lx, n = 11 for controls, n = 9 for *Prkg1^BKO^* mutants for all other conditions. ** p<0.01, * p<0.05 as determined by unpaired t-test. LD, light-dark cycle; DD, constant darkness; LL, constant light. (B) Control and *Prkg1^BKO^* mutant mice were subjected to a lighting schedule mimicking chronic jet lag for 18 days (4 h forward phase shift every two days, see diagram on top: black and white bars indicate distribution of dark and light phases). Total wheel-running activity was quantified before the jet lag, directly after and two months after the treatment. n = 11 for controls, n = 9 for *Prkg1^BKO^* mutants. * p<0.05 as determined by unpaired t-test. (C) Daytime activity was determined for control and *Prkg1^BKO^* mutant mice kept in LD 12∶12 of the indicated intensities. Activity recorded during the light phase is plotted as percentage of total activity. n = 5 for both genotypes for 50 and 10 lx, n = 20 for controls and n = 18 for *Prkg1^BKO^* mutants for 400 lx. *** p<0.001, * p<0.05 as determined by unpaired t-test. (D) Control and *Prkg1^BKO^* mutant mice were kept in LD 12∶12. Bouts of inactivity during the activity phase, e.g. the period of more or less continuous activity, were quantified from the actograms. n = 19 for controls, n = 18 for *Prkg1^BKO^* mutants. ** p<0.01, * p<0.05 as determined by unpaired t-test. (E) The average distribution of activity over one day was analyzed for control and *Prkg1^BKO^* mutant mice kept in LD 12∶12. Black and white bars on top depict the distribution of dark and light periods during one day. Data is expressed as average counts/minute over a ten-minute interval. The inset graph shows ZT 0.33–11.33 on a magnified scale. Gray diamonds indicate statistically significant differences between genotypes (p<0.05 as determined by unpaired t-test). n = 19 for controls, n = 18 for *Prkg1^BKO^* mutants; ZT, Zeitgebertime. (F) Relative drinking activity during the light phase of *Prkg1^BKO^* and *Prkg1^SMr^* mice as compared to their respective litter-matched controls. Values are expressed as % of total drinking volume consumed during the light phase. *** p<0.001, ** p<0.01 as determined by unpaired t-test. n = 8 for both *Prkg1^BKO^* mutants and the respective controls, n = 10 for *Prkg1^SMr^* mutants, n = 21 for the respective controls. Note that the absolute drinking volume during 24 hours was not different between genotypes. (G) Representative recordings of drinking activity over six days for all four genotypes. Black and white bars on top depict the distribution of dark and light periods during the recording. For better orientation, those parts of the diagrams recorded during darkness are additionally shaded.

An important cue to determine the length of a day is the lights off-signal, which, in nocturnal animals, usually coincides with the onset of activity. Therefore we tested how well *Prkg1^BKO^* mice entrained to this cue. We found that these mutants had a less precise onset of wheel-running activity (Fig. 5A) as compared to control mice, which is in accordance with the observed reduction in chi-square periodogram amplitude and a weaker oscillator. This phenomenon was observed for light intensities of 400 and 50 lux but disappeared when only 10 lux were employed, probably due to difficulties in distinguishing between light and dark phase already in control animals (Fig. 5A). Under constant conditions (DD and LL), the two strains also showed comparable onset errors (Fig. 5A). Because of the differences in onset errors in LD, we tested how *Prkg1^BKO^* mutant and control mice reacted to chronic jet lag (4 hour advance every 2 days). Under these conditions, a constant re-adaptation to the changes in lighting schedule is required, which is very stressful for animals with a weak oscillator. Interestingly, total activity of mutants was reduced directly after chronic jet lag, which might be a sign of a higher stress level caused by the irregular lighting schedule, but went back up to control levels when the mice were kept again in a regular LD cycle (Fig. 5B), consistent with a recovery under steady conditions. These observations suggest that *Prkg1^BKO^* mutant mice are not able to determine light-dark transitions as precisely as control animals, and that this has behavioral consequences in a jet lag protocol.

### Activity and rest phases are re-distributed in *Prkg1* mutant mice

The imprecision of determination of light offset in *Prkg1^BKO^* mice caused a higher extent of wheel-running activity during the light phase and thus was reflected in an increase of daytime activity at 400 lux and 50 lux (Fig. 5C), which suggests less sleep in the rest (light) phase. In accordance with this, in the activity period (dark phase), the number of longer rest bouts of 30 minutes to two hours was higher (Fig. 5D), supporting the notion that *Prkg1^BKO^* mice show an altered sleep distribution (Fig. 2). Wheel-running activity was increased during the light phase and decreased during the dark phase in the mutants (Fig. 5E). These findings parallel our observation that *Prkg1^SMr^* mice display more wakefulness in the light period (Fig. 2A, baseline).

We finally chose a complementary approach that allowed us to analyze circadian rhythmicity and basal entrainment to light-dark cycles without the problems associated with the reduced wheel running activity of *Prkg1^SMr^* mice. To this end, we monitored drinking activity of both *Prkg1^BKO^* and *Prkg1^SMr^* mice and their respective controls in LD 14∶10. Both mutants were found to consume a significantly higher percentage of their total water intake during the light phase, while total drinking volume was not different between genotypes (Fig. 5F, G). These observations clearly confirm the findings of sleep and wheel running analysis, namely the notion that PRKG1 deficiency in the nervous system alters the activity pattern in favor of more daytime activity.

## Discussion

In this study we report an altered distribution of sleep and wakefulness as well as of wheel-running and drinking activity in mice lacking functional PRKG1 in the brain. These results could be confirmed independently using two different mouse models, one in which *Prkg1* expression was abolished selectively in the nervous system and one in which it was restricted to smooth muscle cells. Moreover, mutants displayed less REM sleep and a more fragmented NREMS, a phenotype that has also been observed in a rat model of sleep fragmentation [28], as well as a drastic decrease in delta power under baseline conditions.

Although not all parameters were determined in both strains, those analyses performed in parallel indicate that their phenotype is largely identical: Neither of the mutants showed an altered internal period length in DD, and the differences observed in chi square periodogram amplitude as well as in drinking behavior were the same for both mutant strains compared to their respective controls. Furthermore, results for sleep and circadian rhythms were in good accordance. There was no difference in the total amount of sleep, wakefulness, drinking or wheel-running activity, only their distribution over the 24 hours of a day was changed in a way that indicates more activity, or wakefulness, during the day and more sleep, or rest, during the night.

Previous findings about the involvement of NO and cGMP in the regulation of sleep and wakefulness and sleep homeostasis are not fully conclusive and partly contradictory. NO has been reported to promote sleep in rats [29], and injection of an NO donor caused an increase in NREMS [30]; in cats, however, it also appears to be required for arousal [31]. Inhibition of NO synthase (NOS) by L-NAME has been described to reduce both NREMS and REMS in rats [32]; however, also the exact opposite effect has been reported [33]. The specific neuronal NOS (nNOS) inhibitor, 7-nitro-indazole, has been demonstrated to decrease REMS exclusively [33], to reduce both REMS and NREMS as well as EEG amplitude [34], or to have most of all an effect on NREMS [35], respectively. Inhibition of soluble guanylyl cyclase, which generates cGMP upon activation by NO, promoted NREMS and suppressed REMS [36].

Divergent findings might be due to variances in times of treatment [37] and/or drug dose [30], [35]. In addition, it is important to note that not all effects of NO must be mediated via cGMP and PRKGs. For instance, NO can signal in a cGMP-independent manner via S-nitrosylation of target proteins [38]. In any case, two of our main observations in mice deficient in PRKG1 in the brain, namely reduced REM sleep and EEG amplitude, have been made before in animals with impaired NO signaling [32]–[34], implicating that the NO-cGMP-PRKG1 pathway might indeed be involved in sleep regulation. Further support for this hypothesis comes from a study in mutant mice, which revealed that abolition of nNOS, but not inducible NOS (iNOS), reduced REMS in mice [39]. Given that under normal conditions, iNOS is not expressed in the brain (reviewed in [40]), a sleep-regulating pathway involving both nNOS and PRKG1 might be envisaged.

Delta power under baseline conditions was reduced in *Prkg1^SMr^* mice, which also has been described previously after L-NAME treatment [37]. At the same time, more short NREMS episodes were observed in the mutants. The prevalence of EEG delta oscillation is thought to reflect the depth of NREMS and therefore to correlate negatively with sleep fragmentation [41]. Marked increases in the relative contribution of fast oscillations (39.0–60.25 Hz) to the NREMS EEG, as those observed in *Prkg1^SMr^* mice (Fig. S1), have also been associated with a more disturbed sleep and higher levels of cortical arousal during sleep [42].

Based on delta power, sleep homeostasis does not appear to be altered in *Prkg1^SMr^* mice because the drastic reduction in delta power present under baseline conditions was no longer seen after sleep deprivation. However, the marked increase in rebound sleep (both REMS and NREMS), which was especially pronounced immediately after sleep deprivation, suggests a role for PRKG1 in these aspects of homeostatic sleep regulation.

Inhibition of NO signaling in the brain has been reported to have the opposite effect, namely a decrease in sleep rebound, in rats [5], [6]; for mice, however, no data is available. It is conceivable that the contrasting observations in mice and rats reflect species-specific differences. Alternatively, the reduced capacity of *Prkg1^SMr^* mice to sustain long bouts of wakefulness might contribute to the lower levels of EEG delta power reached in these mice because sleep need does not substantially accumulate when sleep is initiated after only a few minutes of wakefulness. Thus, the considerable differences in delta power under baseline conditions might be caused not by changes in the sleep homeostat, but by changes in wake consolidation, which are also confirmed by the circadian data, namely the observation that there are more long rest bouts during the activity phase.

Inhibition of PRKGs has been shown to abolish phase advances or to cause spontaneous phase delays in hamster and rat [43], [44]. However, these effects appeared to be mediated by PRKG2 rather than PRKG1 [14]. The absence of a resetting phenotype in *Prkg1* mutant mice might also be explained by species-specific differences. Mice deficient for nNOS or endothelial NOS (eNOS), e.g., the two NOS species expressed in the brain under non-pathological conditions, were found to display normal phase shifts and an internal period length comparable to their respective controls [45], [46]. NO signaling appears to have a stabilizing effect on the oscillator, though, because treatment with NO donors has been shown to revert the aging-induced decline of circadian rhythms [47]. In agreement with this, treatment of mice with sildenafil, a phosphodiesterase inhibitor which causes sustained cGMP-mediated signaling, improves adaptation after jet lag [48]. These results parallel our observations that, in the absence of PRKG1, mice do not tolerate chronic jet lag as well as control animals.

The sleep data indicates that, in the absence of PRKG1, both sleep and wakefulness are more fragmented and that their distribution over the 24 hours of a day is altered. In accordance with the sleep phenotype, wheel-running and drinking analysis revealed increased activity of *Prkg1* mutants during the day, when rodents are usually not active. A higher fragmentation could also be observed for the activity distribution during the active phase in that *Prkg1^BKO^* animals showed more long rest bouts (Fig. 5D). This is in agreement with the accumulation of more REM sleep in mutant mice in the first half of the dark phase (Fig. 2B), a period during which mice are normally active most of the time and the biggest part of their total wheel-running activity is observed (Fig. 4A, C).

It has recently been shown that alterations of the dynamics of the sleep homeostat can also alter the distribution of sleep [49]. As discussed above, the reduced levels of EEG delta power during baseline might be a consequence of the changes in the distribution of sleep in *Prkg1* mutant mice. The fact that we did observe differences in the compensatory rebound in sleep time after sleep deprivation, however, suggests that PRKG1 also affects at least some aspects of sleep homeostasis. The contribution of this effect to the pronounced reduction in sleep and wake quality observed during baseline remains to be quantified.

Taken together, we present evidence that abolition of PRKG1 in the nervous system distinctly alters the sleep-wake or rest-activity distribution in mice. The reduction in the diurnal amplitude of these overt behaviors is likely to be due to a reduction of the circadian output strength, a hypothesis that is supported by the effects of *Prkg1* deficiency on circadian rhythm amplitude under constant conditions. Especially, the inability of *Prkg1*-deficient mice to sustain long periods of wakefulness is consistent with the notion that the SCN primarily promotes wakefulness, as lesion studies suggest [50], [51].

## Materials and Methods

### Mice

The generation of mice carrying a conditional loxP-flanked (“floxed”) *Prkg1* allele (L2) or a recombined *Prkg1* null allele (L−; see Fig. 1A) and their genotyping by PCR has been described [52]. Mice with modified *Prkg1* alleles were crossed with Nes-Cre mice [22] to generate conditional *Prkg1* mutants lacking PRKG1 in the brain (*Prkg1^BKO^* mice; genotype: *Prkg1* ^L−/L2^; Nes-Cre^tg/0^) and litter-matched control mice (genotype: *Prkg1* ^+/L2^; Nes-Cre^tg//0^). The Nes-Cre transgene was detected by PCR analysis with *cre*-specific primers [53]. To monitor the recombination pattern of the Nes-Cre line, it was crossed with ROSA26 Cre reporter (R26R) mice [23], which express β-galactosidase upon Cre-mediated recombination of a *lacZ* reporter gene. The *Prkg1* smooth muscle (SM) rescue (*Prkg1^SMr^*) mice, which express an SM-specific *Prkg1* transgene on a *Prkg1* ^L−/L−^ background, and litter-matched *Prkg1* ^+/L−^ or *Prkg1* ^+/+^control animals were generated as described [21]. Male mutant and control littermates were used for all behavioral analyses. Experiments in Switzerland were performed according to the state laws of the Cantons of Fribourg and Vaud. Experiments performed in Germany were done in accordance with German animal protection law.

### Immunostaining and X-Gal staining

Expression of PRKG1 was detected in brain extracts by Western blotting and in paraffin sections by immunohistochemistry with a polyclonal antibody against recombinant PRKG1 (PRKG1 common antibody) as described previously [18]. β-galactosidase activity was detected by staining brains (cut into halves or cryosectioned) with 5-bromo-4-chloro-3-indolyl b-D-galactoside (X-Gal) as reported [54].

### Analysis of sleep/EEG

#### Animals and housing conditions

Adult male *Prkg1^SMr^* and control mice (n = 7 for both) of 14–16 weeks of age were used in this study. After surgery, mice were kept individually in polycarbonate cages (31×18×18 cm) with food and water available *ad libitum*, and maintained on a 12 h light–12 h dark cycle (LD 12∶12; lights on at 9:00 AM) at an ambient temperature of 24.5–25.5°C.

#### Surgery

Electroencephalogram (EEG) and electromyogram (EMG) electrodes were implanted under deep anaesthesia with a mixture of ketamine and xylazine (ip, 75 and 10 mg/kg, respectively, at a volume of 8 µl/g). Two gold-plated miniature screws (diameter 1.1 mm) that served as EEG electrodes were screwed into the cranium over the right cerebral hemisphere, in a fronto-parietal position (according to [55]). Four additional anchor screws were implanted; one over the right hemisphere and three over the left hemisphere. Two semi-rigid gold wires served as EMG electrodes and were inserted between two neck muscles. The EEG and EMG electrodes were soldered to a connector and the anchor screws were cemented to the skull. 4 to 8 days of recovery from surgery were allowed before animals were connected to the recording leads. A minimum of 6 adaptation days (or 10 including recovery from surgery) was scheduled before data collection.

#### Experimental protocol

EEG and EMG signals were recorded continuously for 2 days starting at light onset (0 h). The first day (0–24 h) served as baseline. Starting at light onset of the second day animals were kept awake for 6 h (sleep deprivation: 24–30 h) by gentle handling [25]. The remaining 18 h of the second day (30–48 h) were considered recovery.

#### Data analyses

The analogous EEG and EMG signals were digitized at 2000 Hz and subsequently stored at 200 Hz on hard disc. The EEG was subjected to a discrete-Fourier transformation (DFT) yielding power spectra (range: 0.25–100 Hz, resolution: 0.25 Hz, window function: hamming; spectral analysis limited to the 0.75–45.0 Hz range for the current report) for consecutive 4-s epochs. Hardware (EMBLA™) and software (Somnologica-3™) were purchased from Medcare/Flaga (Island). Based on the EEG and EMG signals, the animal's behavior was classified either as REMS, NREMS, or wakefulness, for consecutive 4-s epochs according to standard criteria [55]. States were scored by visual inspection of the EEG and EMG signals displayed on a PC monitor. 4-s epochs containing EEG artifacts were marked so they could be excluded from EEG spectral analyses.

Amount and distribution of NREMS, REMS, and wakefulness were calculated for 1, 12, and 24 h intervals. The baseline distribution of sleep was further analyzed by expressing hourly values of the three behavioral states as % of the total time spent in each state during baseline ( = 100%). Subsequently, hourly percentages were accumulated and genotype differences of the resulting curves calculated to assess the times at which the two distributions deviated. The recovery time course for sleep time lost during the sleep deprivation was calculated by contrasting the hourly values of NREMS and REMS observed during recovery (30–48 h) to the values observed during the corresponding baseline hours (6–24 h). The recovery-baseline differences were accumulated to examine the recovery time course. NREMS quality during baseline was assessed by determining its fragmentation. For the number of NREMS episodes <1 min (i.e., 15 or less consecutive 4-s epochs scored as NREMS) and >1 min were calculated [25]. Both variables were expressed per hour of NREMS to correct for eventual individual differences in time spent in this state.

EEG frequency content was analyzed using the DFT. For each behavioral state an EEG spectral profile (0.75–45 Hz) was constructed by averaging all artifact free 4-s epochs scored as that state. An additional inclusion criteria was that the 4-s epoch under consideration should be immediately preceded and followed by a 4-s epoch that was of the same state and equally artifact free. Spectra were calculated as absolute values, in units of micro-volts square per 0.25 Hz, or as relative values to correct for inter-individual differences in the absolute EEG level. The latter analysis quantifies the relative contribution of each frequency to the overall EEG signal during each of the three behavioral states. All values are expressed relative to the weighted mean average of total EEG power in all vigilance states [55].

EEG delta power during NREM sleep was calculated by averaging power density in the frequency bins from 1 to 4 Hz. Values were individually normalized by expressing them as a percentage of the mean delta power over 4-s epochs scored as NREMS in the last 4 h of the baseline light period; i.e., a time at which the lowest average levels are reached during baseline. The time course of EEG delta power in NREMS was assessed by dividing the 12 h light and dark periods into 12 (baseline light period; 0–12 h), 8 (recovery light 30–36 h and dark period 36–48 h), or 6 (for the baseline dark period; 12–24 h) intervals to which an equal number of 4-s epoch scored as NREMS contributed within individual mice (i.e., percentiles). The same analysis was performed to assess the dynamics of EEG power in NREMS in the 1.0–2.25 Hz and 2.5–4.0 Hz frequency ranges and for the 1.0–2.25/2.5–4.0 Hz power ratio.

All statistical analyses were done using SAS (SAS Institute Inc.; version 9.1). All graphics including non-linear fitting (for illustration purposes) were done using SigmaPlot (SyStat Software, Inc.; version 9).

### Wheel-running

#### Animals

Animals used were adult male *Prkg1^SMr^* and control mice (n = 6 for both) and adult male *Prkg1^BKO^* mice (n = 18) and their respective controls (n = 20) between 7–16 weeks of age at the beginning of the experiments.

#### Data collection and analysis

Analysis of locomotor activity parameters was done by monitoring wheel-running activity as described in [56] using the ClockLab software (Actimetrics) for all subsequent calculations. Briefly, for the analysis of free-running rhythms, animals were entrained to LD 12∶12 and subsequently released into constant darkness (DD) or constant light (LL). Internal period length (τ) was determined from a regression line drawn through the activity onsets of 10 days of stable rhythmicity under constant conditions. Total and daytime activity as well as activity distribution profiles and chi square periodograms were calculated using the respective inbuilt functions of the ClockLab software. Onset errors were determined manually from the actograms and refer to the difference between lights off and the actual onset of activity in LD and to the deviation of the actual onset from a regression line drawn through 10 consecutive onsets in LL/DD. Length and frequency of rest bouts during the activity phase were also evaluated manually from the actograms of animals kept in LD 12∶12. For chronic jet-lag, animals were subjected to a lighting schedule mimicking a 4 h-forward shift every 2 days for 18 days. Phase shifts were determined according to the Aschoff Type II protocol [57]. Animals were entrained to LD 12∶12, subjected to a light pulse (15 min, 400 lux) at Zeitgebertime (ZT) 14 and subsequently released into DD for 14 days. They were again entrained to LD12∶12 and the procedure was repeated for a light pulse at ZT22. To monitor the effect of a simple LD-DD-transition, animals were entrained to LD 12∶12 and released into DD without prior adminstration of a light pulse. For the calculation of phase shifts, a regression line was drawn through 10 consecutive onsets in DD; the first 2 days in DD were regarded as transition and not taken into account. Phase shifts were expressed as the differences between the regression lines (and thus the hypothetical onsets of activity) on the first day of DD after a normal LD-DD transition and after a light pulse. Statistical analysis was perfomed using Prism 4 for Macintosh (Graph Pad software, Inc.; version 4.0a).

### Open-field test

#### Animals

Animals used were 9 months-old male *Prkg1^SMr^* (n = 5) and control mice (n = 6).

#### Data collection and analysis

General locomotor activity was assessed using an infrared beam-operated system (Institute of Physiology, University of Fribourg, Switzerland) in which a standard cage (16×22.5 cm) was placed inside a frame containing 96 infrared LEDs in 2 levels of 24×24 LEDs. The first level was at a height of 2.8 cm, the second level at a height of 5.5 cm; horizontal distance between LEDs was 8.5 mm on the long side and 6.25 mm on the short side of the cage. The test was performed in cages with Whatman paper as bedding so as not to disturb the readings. Animals were allowed to get used to the absence of bedding for one day prior to the experiment and were subjected to the test on the next day between 10 AM and 1 PM (ZT 4–7). At the beginning of the test session, animals were placed in the middle of the test cage, and locomotor activity (in the form in infrared beam breaks) was recorded for 30 minutes using a Visual Basics-based software (ActivityX16, provided by Laurent Monney, Institute of Physiology, University of Fribourg, Switzerland). Data was transmitted as serial information using an RS232 protocol and evaluated using a C sharp-based software (Read Activity Results File version 1.4.4, also provided by Laurent Monney, Institute of Physiology, University of Fribourg, Switzerland). Total distance travelled, mean speed and percentage of total time spent active were calculated for each animal.

### Drinking activity

#### Animals

Animals used were 6–13 week-old male *Prkg1^SMr^* mice (n = 10) and litter-matched control mice (n = 21), and 10–36 week-old male *Prkg1^BKO^* mice (n = 8) and litter-matched control mice (n = 8).

#### Data collection and analysis

Drinking activity and volume of individually housed mice were monitored continuously using a computerized system (TSE Technical & Scientific Equipment GmbH, Bad Homburg, Germany). Animals were monitored for 4–6 days with food and water available *ad libitum*, and maintained on an LD 14∶10 cycle (lights on at 6:00 AM).

## Supporting Information

Figure S1 EEG spectral composition of wakefulness (W), non-Rapid-Eye-Movement sleep (NREMS, N), and REMS (R) during baseline. (A) Absolute EEG power density for W (gray), N (blue), and R (red) in control (upper panel) and Prkg1SMr (middle panel) mice. The lower panel depicts the % Prkg1SMr/control difference spectra. Genotype affected absolute EEG spectra of NREMS only [2-way ANOVA with factors ‘genotype’ (P = 0.37, 0.0072, and 0.44) and ‘EEG frequency’ (repeated measures, 0.75–45 Hz: P<0.0001) and their interaction (P = 0.18, 0.0009, and 0.50); P-values for W, N, and R, respectively]. Horizontal bars at the bottom connect frequency bins in which values differed between genotypes (P<0.05; post-hoc 2-sided t-tests), color coded according to behavioral state. (B) Relative EEG spectra. Within each mouse, behavioral state, and frequency bin, EEG power density was expressed as a % of the total EEG power over all frequency bins (0.75–45 Hz) and behavioral states in baseline. This EEG reference values was weighted so that an equal number of 4 s of each state contributed to the total in all mice (see Methods). Normalizing reduces the variance in the data due to inter-individual differences in EEG signal strength but precludes the analysis of genotype effects on absolute EEG values. Analyses-of-variance indicated that also for these normalized EEG spectra, genotype altered the spectral composition of NREMS only [2-way ANOVA with factors ‘genotype’ (P = 0.12, 0.084, and 0.19) and ‘EEG frequency’ (repeated measures, 0.75–45 Hz: P<0.0001) and their interaction (P = 0.093, 0.011, and 0.32); P-values for W, N, and R, respectively]. During NREMS, relative EEG power density in Prkg1SMr mice (black line) was reduced in the low delta (0.75–2.0 Hz) and increased in the gamma (39.0–60.25 Hz) frequency range compared to control mice (gray line). Gray horizontal bars at the bottom connect frequency bins for which values differed between genotypes (P<0.05; post-hoc 2-sided t-tests).
(1.33 MB TIF)

Figure S2 Frequency distribution of waking (W, top), Rapid-Eye-Movement sleep (REMS, R, bottom), and non-REMS (NREMS, N, middle panels) episode duration in baseline. Mean (+SEM) number of episode expressed per hour of the respective behavioural state for nine consecutive time bins (4 s, 8–12 s, 16–28 s, 32–60 s, 64–124 s, 128–252 s, 256–508 s, 512–1020 s, and >1024 s; left panels) and the time spent in each time bin (right panels; % of the individual total time spent in each behavioural state over the 24 h baseline). The frequency distribution of waking and NREMS episode length was affected by genotype [2-way ANOVA with factors ‘genotype’ (P = 0.033 and 0.017) and ‘episode duration’ (repeated measures, 9 bins; P<0.0001) and their interaction (P = 0.15 and 0.0065); P-values for W and N, respectively]. The distribution of the relative amount of time spent awake or in NREMS also varied with genotype (interaction between factors ‘genotype’ and ‘episode duration’: P<0.0001 and 0.035 for W and N, respectively). REMS episode duration was not affected by genotype. Red asterisks indicate significant genotype differences (P<0.05; post-hoc t-tests).
(1.70 MB DOC)

Figure S3 Time course of EEG delta power. Given the frequency specific effects of genotype on EEG power density within the delta frequency range (see Fig. 2 and Fig. S1), the time course of EEG delta power was analyzed separately for slow (1.0–2.25 Hz) and fast (2.5–4.0 Hz) delta frequencies. (A) Mean (±1SEM) relative levels of slow (closed symbols) and fast (open symbols) EEG delta power for the 48 h of the experiment (control: upper panel, Prkg1SMr: lower panel). Values within each frequency band were expressed as % of their respective levels reached between baseline hours 8–12 h (see Methods). Changes in slow delta power respond less accurately to changes in sleep-wake distribution as compared to the power in the faster frequencies. This is especially clear in Prkg1SMr mice in which the typical decrease in the light period and increase in the dark period was absent in slow delta power while changes in activity in the faster delta frequencies resembled more that in control mice most notably during recovery. Filled and open diamonds at the bottom indicate times at which values significant differed between genotypes for the slow and fast delta bands, respectively (P<0.05; post-hoc 2-sided t-tests). [3-way ANOVA with factors ‘genotype’? (P = 0.27 and 0.038), ‘SD’ (recovery vs. baseline; repeated measures; P = 0.091 and 0.038) and ‘time’ (30–48 vs. 0–6 and 12–24; repeated measures: P<0.0001); interactions ‘genotype’×‘SD’ (P = 0.038 and 0.33), ‘genotype’×‘time’ (P = 0.095 and 0.096), ‘SD’×‘time’ (P = 0.0017 and <0.0001); P-values for slow and fast delta power, respectively]. B: Time course of the fast-to-slow delta power ratio. This ratio, which is known to increase immediately after long period of wakefulness ([58]; see after SD), was significantly higher in Prkg1SMr due to the larger suppression of slow delta activity compared to fast delta (see Fig. 2). [3-way ANOVA with factors ‘genotype’ (P = 0.0043), ‘SD’ (recovery vs. baseline; repeated measures; P = 0.0004), and ‘time’ (30–48 vs. 0–6 and 12–24; repeated measures: P<0.0001); interactions ‘genotype’×‘SD’ (P = 0.018), ‘genotype’×‘time’ (P = 0.12), ‘SD’×‘time’ (P = 0.0017)]. Gray diamonds at the bottom indicate times at which ratios significant differed between genotypes (P<0.05; post-hoc 2-sided t-tests). In all panels, gray areas denote the dark periods. Recovery values labeled red are significantly different from corresponding baseline values (with respect to sleep onset; P<0.05; post-hoc 2-sided paired t-tests).
(1.29 MB TIF)

Figure S4 Prkg1SMr mutant mice do not display differences in general locomotor activity. General locomotor activity parameters of control and Prkg1SMr mutant mice were determined by monitoring their activity in an open field using a system based on infrared beam breaks during 30 minutes. Total distance travelled (A), mean speed (B) and the percentage of time during which the mouse was active (C) were calculated for each animal. n = 6 for controls, n = 5 for Prkg1SMr mutants.
(0.13 MB TIF)

Figure S5 Clock resetting in Prkg1BKO mutant mice. Phase-shifting properties of control and Prkg1BKO mutant mice were assessed using an Aschoff Type II protocol. Mice were entrained to LD 12∶12, subjected to 15-minutes light pulses at the indicated ZTs and subsequently released into DD. They were additionally subjected to an LD-DD transition without prior administration of a light pulse. Phase shifts are expressed as the difference between the onsets of activity observed on the first day after the light pulse and the first day of DD without light pulse. n = 11 for controls, n = 9 for Prkg1BKO mutants.
(0.10 MB TIF)
