# Supplementary figures and images for: cGMP-Dependent Protein Kinase Type I Is Implicated in the Regulation of the Timing and Quality of Sleep and Wakefulness
Source: PLoS One. 2009 Jan 21;4(1):e4238. doi: 10.1371/journal.pone.0004238 (PMC2617781; doi:10.1371/journal.pone.0004238)

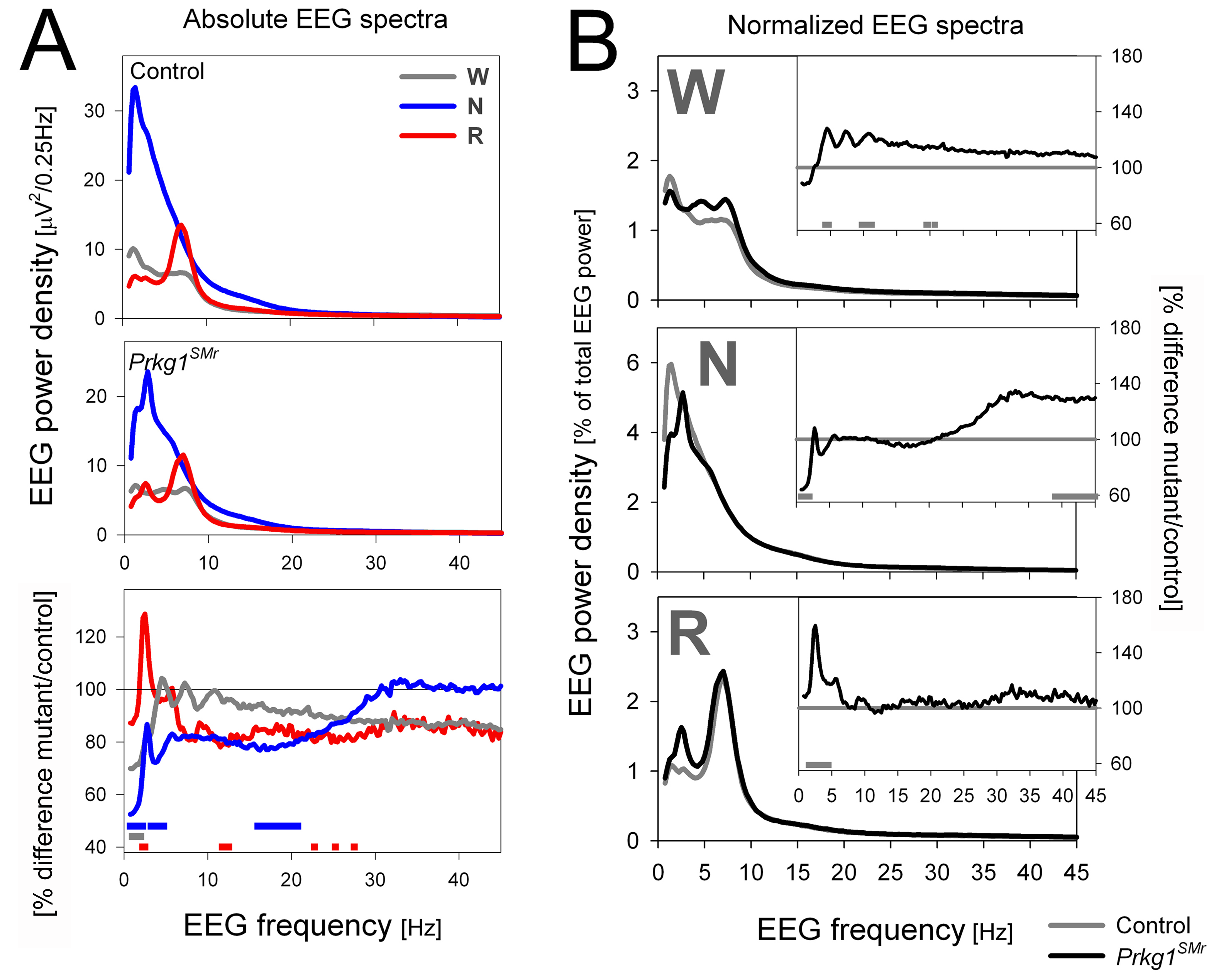

Supplement: Figure S1 — EEG spectral composition of wakefulness (W), non-Rapid-Eye-Movement sleep (NREMS, N), and REMS (R) during baseline. (A) Absolute EEG power density for W (gray), N (blue), and R (red) in control (upper panel) and Prkg1SMr (middle panel) mice. The lower panel depicts the % Prkg1SMr/control difference spectra. Genotype affected absolute EEG spectra of NREMS only [2-way ANOVA with factors ‘genotype’ (P = 0.37, 0.0072, and 0.44) and ‘EEG frequency’ (repeated measures, 0.75–45 Hz: P<0.0001) and their interaction (P = 0.18, 0.0009, and 0.50); P-values for W, N, and R, respectively]. Horizontal bars at the bottom connect frequency bins in which values differed between genotypes (P<0.05; post-hoc 2-sided t-tests), color coded according to behavioral state. (B) Relative EEG spectra. Within each mouse, behavioral state, and frequency bin, EEG power density was expressed as a % of the total EEG power over all frequency bins (0.75–45 Hz) and behavioral states in baseline. This EEG reference values was weighted so that an equal number of 4 s of each state contributed to the total in all mice (see Methods). Normalizing reduces the variance in the data due to inter-individual differences in EEG signal strength but precludes the analysis of genotype effects on absolute EEG values. Analyses-of-variance indicated that also for these normalized EEG spectra, genotype altered the spectral composition of NREMS only [2-way ANOVA with factors ‘genotype’ (P = 0.12, 0.084, and 0.19) and ‘EEG frequency’ (repeated measures, 0.75–45 Hz: P<0.0001) and their interaction (P = 0.093, 0.011, and 0.32); P-values for W, N, and R, respectively]. During NREMS, relative EEG power density in Prkg1SMr mice (black line) was reduced in the low delta (0.75–2.0 Hz) and increased in the gamma (39.0–60.25 Hz) frequency range compared to control mice (gray line). Gray horizontal bars at the bottom connect frequency bins for which values differed between genotypes (P<0.05; post-hoc 2-sided t-tests). (1.33 MB TIF) [file pone.0004238.s001.tif]

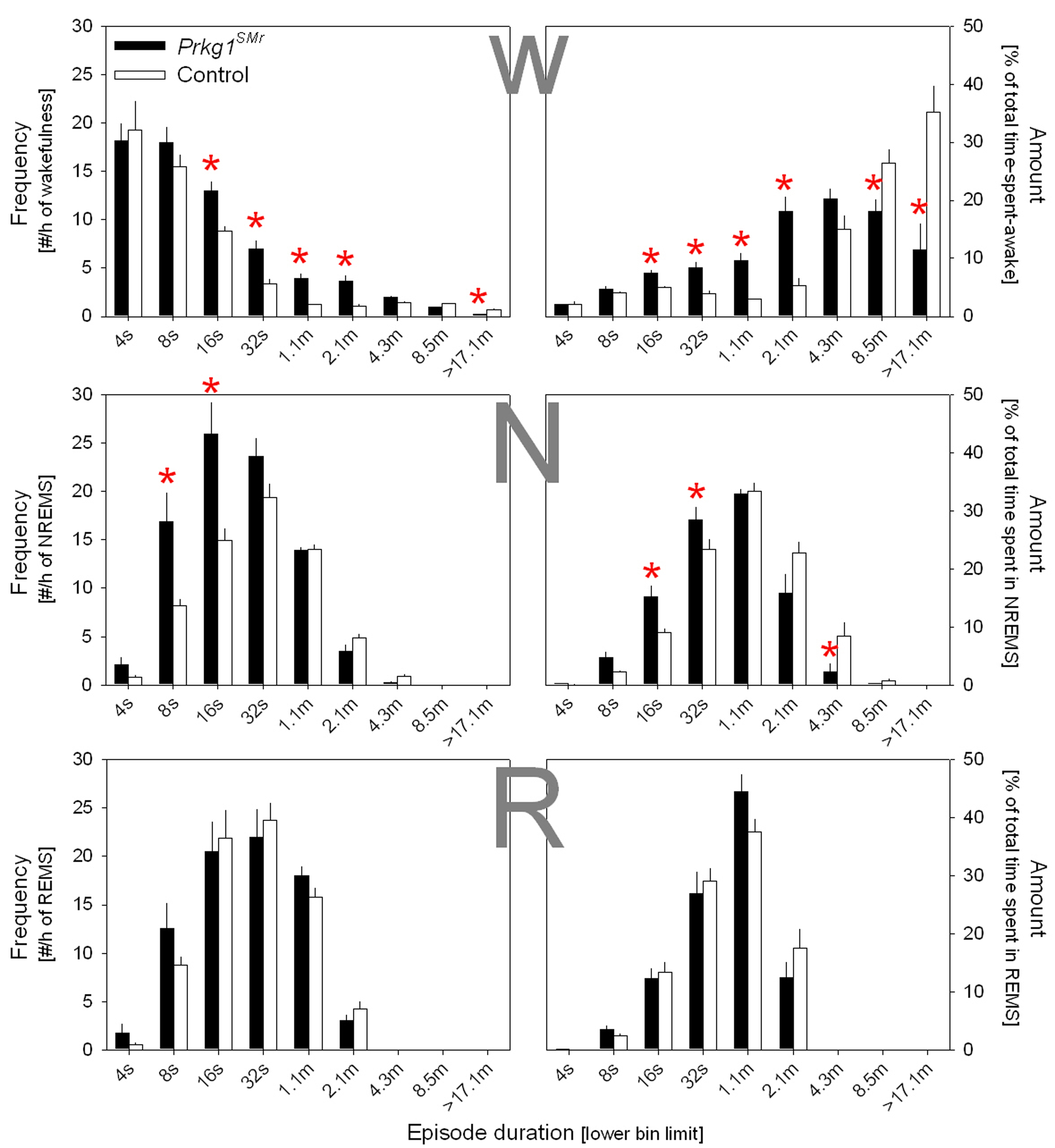

Supplement: Figure S2 — Frequency distribution of waking (W, top), Rapid-Eye-Movement sleep (REMS, R, bottom), and non-REMS (NREMS, N, middle panels) episode duration in baseline. Mean (+SEM) number of episode expressed per hour of the respective behavioural state for nine consecutive time bins (4 s, 8–12 s, 16–28 s, 32–60 s, 64–124 s, 128–252 s, 256–508 s, 512–1020 s, and >1024 s; left panels) and the time spent in each time bin (right panels; % of the individual total time spent in each behavioural state over the 24 h baseline). The frequency distribution of waking and NREMS episode length was affected by genotype [2-way ANOVA with factors ‘genotype’ (P = 0.033 and 0.017) and ‘episode duration’ (repeated measures, 9 bins; P<0.0001) and their interaction (P = 0.15 and 0.0065); P-values for W and N, respectively]. The distribution of the relative amount of time spent awake or in NREMS also varied with genotype (interaction between factors ‘genotype’ and ‘episode duration’: P<0.0001 and 0.035 for W and N, respectively). REMS episode duration was not affected by genotype. Red asterisks indicate significant genotype differences (P<0.05; post-hoc t-tests). (1.70 MB DOC) [file pone.0004238.s002.doc]

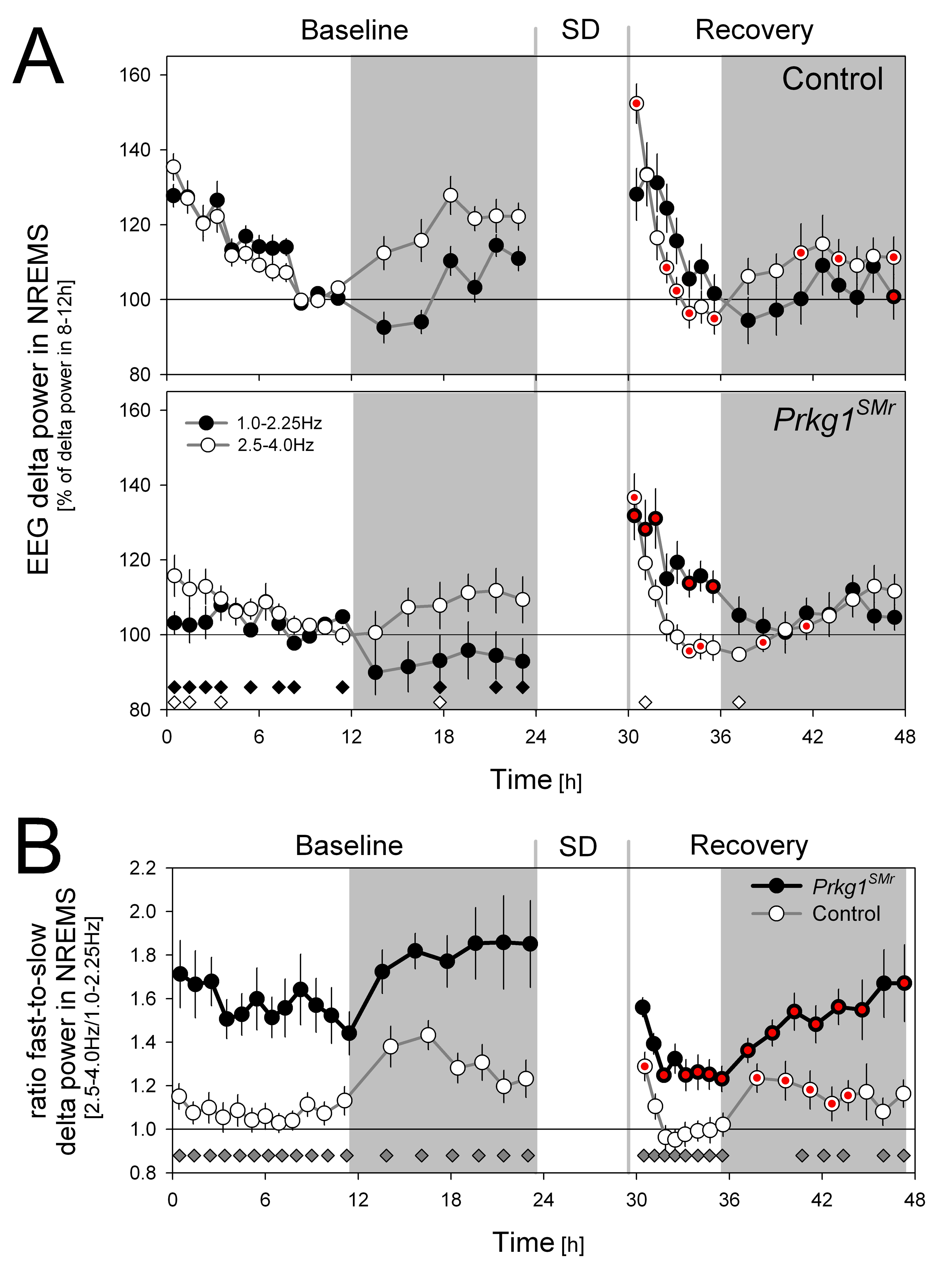

Supplement: Figure S3 — Time course of EEG delta power. Given the frequency specific effects of genotype on EEG power density within the delta frequency range (see Fig. 2 and Fig. S1), the time course of EEG delta power was analyzed separately for slow (1.0–2.25 Hz) and fast (2.5–4.0 Hz) delta frequencies. (A) Mean (±1SEM) relative levels of slow (closed symbols) and fast (open symbols) EEG delta power for the 48 h of the experiment (control: upper panel, Prkg1SMr: lower panel). Values within each frequency band were expressed as % of their respective levels reached between baseline hours 8–12 h (see Methods). Changes in slow delta power respond less accurately to changes in sleep-wake distribution as compared to the power in the faster frequencies. This is especially clear in Prkg1SMr mice in which the typical decrease in the light period and increase in the dark period was absent in slow delta power while changes in activity in the faster delta frequencies resembled more that in control mice most notably during recovery. Filled and open diamonds at the bottom indicate times at which values significant differed between genotypes for the slow and fast delta bands, respectively (P<0.05; post-hoc 2-sided t-tests). [3-way ANOVA with factors ‘genotype’? (P = 0.27 and 0.038), ‘SD’ (recovery vs. baseline; repeated measures; P = 0.091 and 0.038) and ‘time’ (30–48 vs. 0–6 and 12–24; repeated measures: P<0.0001); interactions ‘genotype’×‘SD’ (P = 0.038 and 0.33), ‘genotype’×‘time’ (P = 0.095 and 0.096), ‘SD’×‘time’ (P = 0.0017 and <0.0001); P-values for slow and fast delta power, respectively]. B: Time course of the fast-to-slow delta power ratio. This ratio, which is known to increase immediately after long period of wakefulness ([58]; see after SD), was significantly higher in Prkg1SMr due to the larger suppression of slow delta activity compared to fast delta (see Fig. 2). [3-way ANOVA with factors ‘genotype’ (P = 0.0043), ‘SD’ (recovery vs. baseline; repeated measures; P = 0.0004), and ‘time’ ( [file pone.0004238.s003.tif]

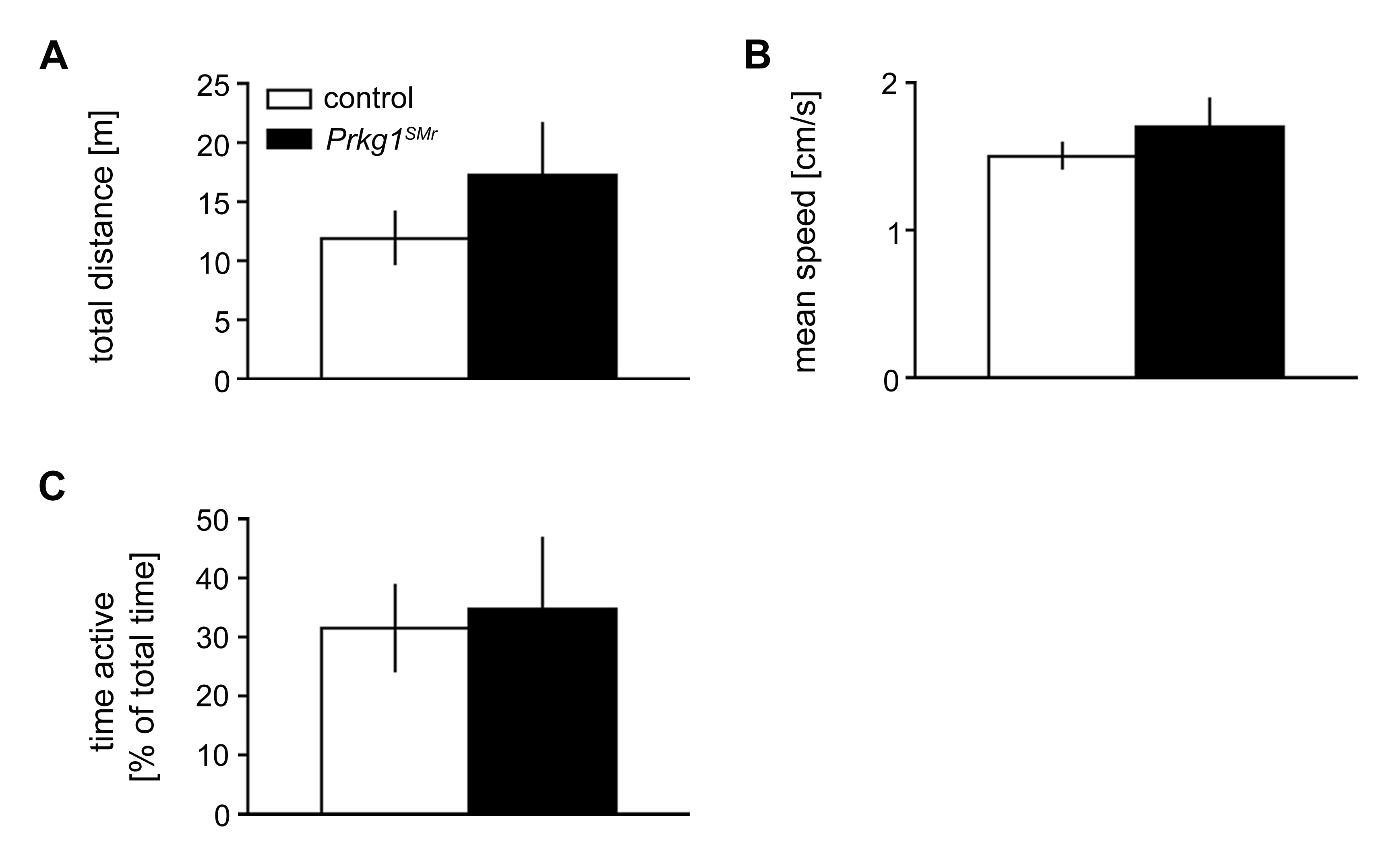

Supplement: Figure S4 — Prkg1SMr mutant mice do not display differences in general locomotor activity. General locomotor activity parameters of control and Prkg1SMr mutant mice were determined by monitoring their activity in an open field using a system based on infrared beam breaks during 30 minutes. Total distance travelled (A), mean speed (B) and the percentage of time during which the mouse was active (C) were calculated for each animal. n = 6 for controls, n = 5 for Prkg1SMr mutants. (0.13 MB TIF) [file pone.0004238.s004.tif]

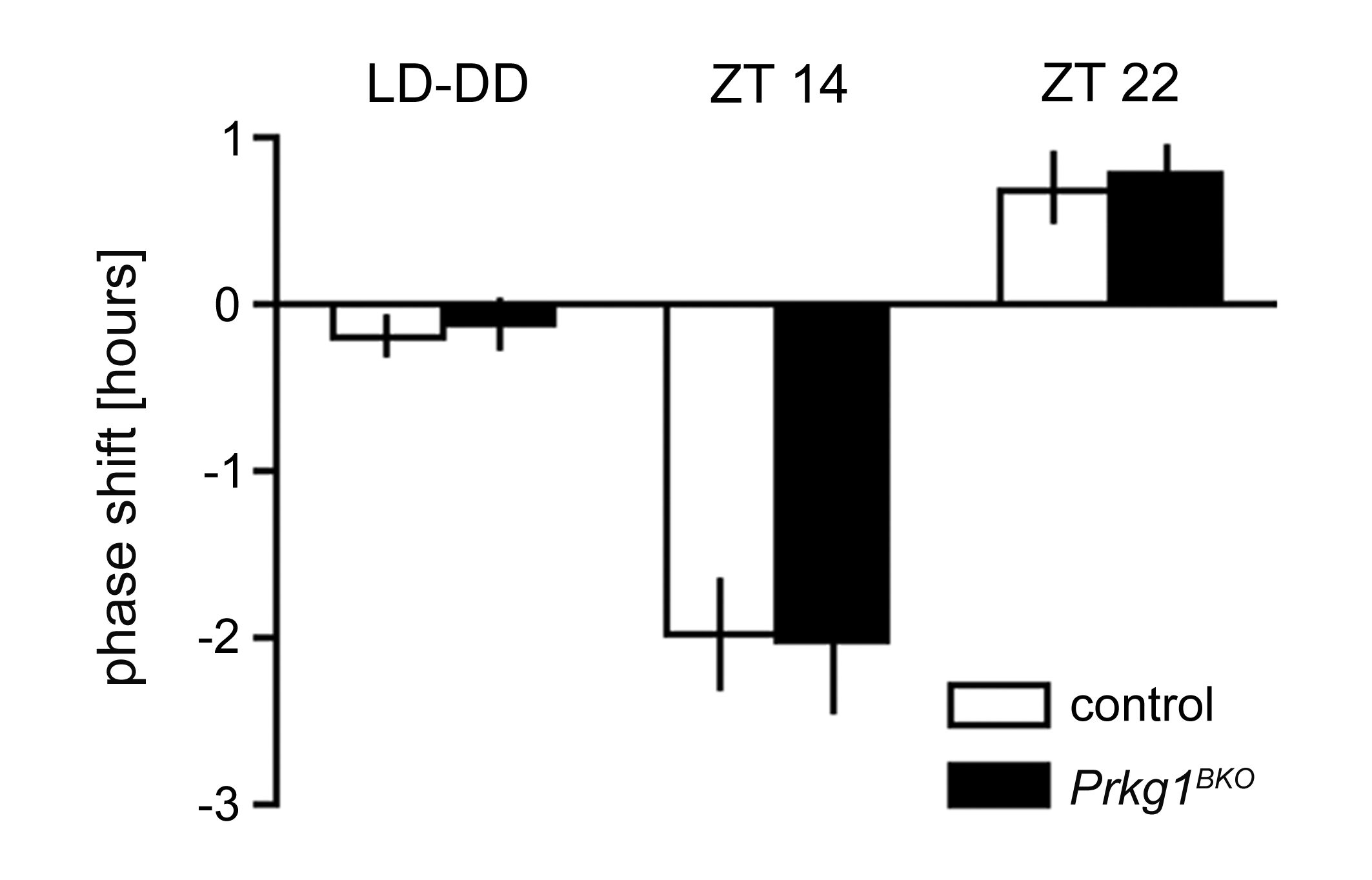

Supplement: Figure S5 — Clock resetting in Prkg1BKO mutant mice. Phase-shifting properties of control and Prkg1BKO mutant mice were assessed using an Aschoff Type II protocol. Mice were entrained to LD 12∶12, subjected to 15-minutes light pulses at the indicated ZTs and subsequently released into DD. They were additionally subjected to an LD-DD transition without prior administration of a light pulse. Phase shifts are expressed as the difference between the onsets of activity observed on the first day after the light pulse and the first day of DD without light pulse. n = 11 for controls, n = 9 for Prkg1BKO mutants. (0.10 MB TIF) [file pone.0004238.s005.tif]
